# *In vitro* assessment of antibacterial activity in four endodontic sealers against *Staphylococcus aureus* and *Kocuria rhizophila* using agar diffusion test

**DOI:** 10.25122/jml-2022-0337

**Published:** 2023-04

**Authors:** Nibrass Talib Al-Quraine, Jaber Faez Abdulkadhim Al-Ibraheem, Yassir Hammed Edan Zyara, Wasna’a Mohamed Abdulridha

**Affiliations:** 1 Department of Conservative, Faculty of Dentistry, University of Kufa, Najaf, Iraq; 2 Department of Conservative, Faculty of Dentistry, Islamic University, Najaf, Iraq; 3 Department of Physics, Faculty of Science, University of Kufa, Najaf, Iraq

**Keywords:** antibacterial effectiveness, AH26, ZOE, Apexit, EndoRez, ADT – Agar Diffusion Test, BHIB – Brain Heart Infusion Broth, ZOE – Zinc Oxide, TSB – Tryptic Soy Broth

## Abstract

In this in vitro study, we assessed the antibacterial efficacy of four endodontic sealers—resin AH26, EndoRez, calcium hydroxide (Apexit), and pure zinc oxide—against *Enterococcus faecalis*. The agar diffusion test was employed to evaluate the antibacterial efficacy of the sealers in vitro, with distilled water serving as a control. The sealers were prepared following the manufacturer's instructions and placed in wells of 50 agar plates, each inoculated with 15 samples of *Kocuria rhizophila* and *Staphylococcus aureus*. Inhibition zones were assessed after 72, 120, and 168 hours of anaerobic incubation at 37°C for 196 hours. Kruskal-Wallis and Friedman tests were used for data analysis. Positive control plates exhibited bacterial growth in all specified periods. AH26 demonstrated significantly higher antibacterial effectiveness against both bacterium types compared to the other sealers (P<0.01). Pure zinc oxide exhibited moderate antibacterial activity, while Apexit and EndoRez showed the lowest activity against *S. aureus* and no activity against *K. rhizophila*. AH26 had the highest antibacterial effect, and EndoRez had the lowest (P<0.05). In terms of inhibiting bacterial growth, the effectiveness of root canal sealers was ranked as follows: AH26 > Pure Zinc Oxide >Apexit/EndoRez.

## INTRODUCTION

Apical periodontitis symptoms and indications, primarily caused by bacterial infections in the root canal system, are linked to the success or failure of endodontic fillings [1]. According to Komabayashi et al., endodontic failure and persistent apical periodontitis are frequently caused by microbial infections in root canals [2]. Therefore, addressing chronic apical periodontitis requires developing new tools and techniques, such as root canal therapy, apical microsurgery, or dental reimplantation, and their continuous improvement [3]. Various root canal sealer materials are used in endodontic fillings, each with advantages and disadvantages. Sealers are primarily selected based on their sealing ability, adhesive properties, biocompatibility, and antibacterial effectiveness [4]. Root canal sealers play a crucial role in filling spaces that core materials cannot reach, such as bifurcation, apical ramification, and lateral canals [5]. Komabayashi et al. observed that bacteria and fungi persist in dentinal tubules, crevices, and root canals even after thorough root canal cleansing [2]. They also mention that bacteria can enter obstructed root canals if the coronal seal is inadequate. Several bacteriological studies have shown that many gram-positive facultative anaerobic bacteria, such as *Enterococci, Lactobacilli, Streptococci*, and *Actinomyces*, can survive even after root canal therapy [6].

Achieving a compact, fluid-tight seal of the apical end of the root canal is critical for successful root canal therapy, as it prevents irritants from entering and accumulating, which can cause the biological breakdown of the attachment apparatus and treatment failure [1]. Tyagi et al. describes various types of root canal sealers based on their chemistry, including silicon-based, glass-ionomer-based, zinc oxide-based (ZOE), bioceramic-based, resin-based, and MTA-based sealers (Mineral Trioxide Aggregate) [7]. ZOE sealers remain popular due to their low cost, slow setting, antibacterial properties, and ease of use. They contain zinc oxide powder and eugenol liquid, an essential oil derived from cloves. The amorphous gel created by mixing zinc oxide and eugenol is inserted into moist root dentin, forming a hard matrix with the remaining zinc oxide powder [8].

Numerous studies have used antibacterial substances, such as antibiotics [9], nanomaterials [10], and quaternary ammonium salts [11] in root canal sealers. However, most of these studies focused on a single bacterial species (*Enterococcus faecalis*). The aim of this study was to evaluate the antibacterial efficacy of four different endodontic sealers against *Staphylococcus aureus* (*S. aureus*) and *Kocuria rhizophila* (*K. rhizophila*), two types of bacteria isolated from the root canal, at 72, 120, and 168 hours [11].

## Material and Methods

The agar diffusion test (agar-well technique) was used in vitro to assess the antibacterial efficacy of the selected root canal sealers against two reference strains of bacteria. AH26 sealer (Dentsply De Trey, Konstanz, Germany), EndoREZ sealer (Ultradent Products Inc., South Jordan, UT, USA), Apexit sealer (IvoclarVivadent Inc., NY, USA), ZOE sealer (Kemdent work Ltd, England), and distilled water as control were prepared following the manufacturer's instructions. *S. aureus*, a facultative anaerobic bacteria, and *K. rhizophila*, an obligate anaerobic bacteria, were used in this study. Both bacterial strains were maintained in pre-reduced, anaerobically sterilized brain heart infusion broth (BHIB) supplemented with 5.0 mg/L hemin and 0.5 mg/L menadione (Difco Laboratories, Michigan, USA). The inoculum's turbidity, prepared in TSB and BHIB, was adjusted to 0.4 McFarland Standard.

The Mitissalivaris agar plate (MSA; Difco, Michigan, USA) for *S. aureus* and the brucella blood agar plate for *K. rhizophila* were the media utilized for the agar diffusion assay. After inoculating the bacteria with a sterile cotton swab, 4 wells (4 mm depth and 6 mm diameter) were punched into each agar plate and filled with freshly prepared sealers, following the protocol described in a previous study [12]. *K. rhizophila* and *S. aureus* were inoculated onto the agar plates. Agar plates containing both bacteria were incubated at 37°C in an anaerobic atmosphere with 5% CO2, 10% H2, and 85% N2 for one week. Positive control plates contained bacteria without root canal sealant. The diameters of bacterial inhibition zones for each sealer were measured and recorded at 72, 120, and 168 hours, using a diameter cutoff value of 6 mm. Five agar plates were used for each bacterial strain, and all experiments were repeated five times to ensure repeatability.

### Statistical analysis

Statistical analysis was conducted using Statistical Software for Social Sciences (SPSS) version 12.0 (SPSS, Inc., Chicago, IL, USA). Kruskal-Wallis and Friedman tests were employed for variance testing, with a p-value <0.05 considered statistically significant at a 95% confidence interval. Mann-Whitney and Wilcoxon tests were used for multiple comparisons, following the Bonferroni correction of α.

## Results

**Figure 1:**
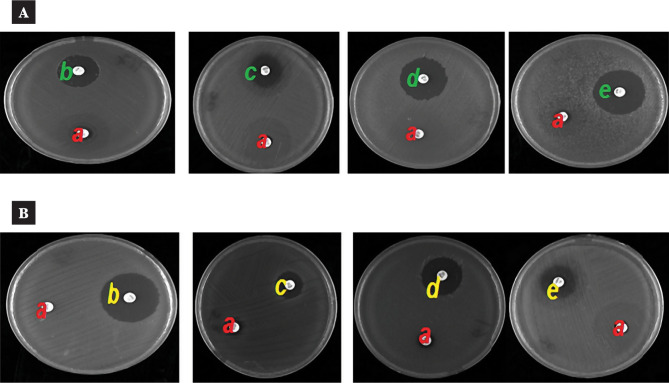
Mean (SD) diameter (in mm) of *S. aureus* and *K. rhizophila* growth inhibition zones for the four root canal sealers in different periods according to ADT; A: *Kocuria rhizophila*, B: *Staphylococcus aureus*; a: distilled water, b: AH26, c: Pure ZOE, d: Apexit, e: EndoRez.

The results are presented in Tables 1-2 and Figure 1 (A-B). All the sealers tested demonstrated antibacterial effectiveness, with significant differences between them (P<0.01). For all the specified time intervals, the AH26 sealer showed more extensive inhibition zones against *S. aureus* and *K. rhizophila* compared to the other sealers (P<0.01). The pure ZOE sealer exhibited moderate antibacterial activity against both bacterial types. In contrast, Apexit and EndoRez displayed the lowest antibacterial effectiveness with *S. aureus* and no antibacterial activity with *K. rhizophila*. Positive control plates showed bacterial growth in all cases. The results indicate that the AH26 sealer had the highest antibacterial effectiveness, while the EndoRez sealer had the lowest effect (P<0.05). Figure 1 presents the ascending order of bacterial growth inhibition zones for the root canal sealers: AH26 > Pure ZOE >Apexit/EndoRez.

**Table 1:** Mean (SD) diameter of *S. aureus* growth inhibition zones for the four root canal sealers.

Material	72 hours Mean±SD	120 hours Mean±SD	168 hours Mean±SD	P-value
**AH26**	36.16±3.68	37.96±6.98	37.96±6.68	0.62
**Apexit**	13.43±0.76	15.10±1.44	15.10±1.44	0.41
**Pure ZOE**	25.5±1.04	26.36±1.20	26.36±1.20	0.07
**EndoRez**	10.40±0.68	12.11±1.20	12.11±1.20	0.37
**Distilled water (control)**	0	0	0	-
**P-value**	<0.01	<0.01	<0.01	-

**Table 2:** Mean (SD) diameter of *K. rhizophila* growth inhibition zones for the four root canal sealers

Material	72 hours Mean±SD	120 hours Mean±SD	168 hours Mean±SD	P-value
**AH26**	41.80±2.17	42.00±2.88	42.00±2.88	0.32
**Apexit**	0	0	0	-
**Pure ZOE**	19.11±2.27	18.69±2.46	18.69±2.46	0.02
**EndoRez**	0	0	0	-
**Distilled water (control)**	0	0	0	-
**P-value**	<0.01	<0.01	<0.01	-

## Discussion

The antibacterial properties of sealers play a crucial role in eliminating residual microorganisms that may have escaped mechanical devices and chemicals, thus enhancing the success rate of endodontic therapy [2]. Various factors such as time determination, solubility, antimicrobials, biocompatibility, sealing ability, and cytotoxicity are all essential to the performance of endodontic sealers [2]. The optimal endodontic sealer should possess longevity, airtightness, biocompatibility, dimensional stability, and antimicrobial effectiveness [1,13]. In this study, ADT was used, and one of the disadvantages of this test is that it does not distinguish between bactericidal properties. The lack of standardization in inoculum density, culture media, sample size, number of specimens per plate, storage conditions, agar viscosity, temperature, and incubation duration can significantly impact the assessment of endodontic sealers' antibacterial effectiveness [14,15]. These parameters should be standardized and integrated to obtain more conclusive results and minimize the influence of in vivo variations. The sensitivity and antibacterial properties of endodontic sealers are influenced by various factors, such as material type, testing method, bacterial species, and time intervals. However, most studies primarily focus on *E. faecalis* when evaluating the antibacterial effectiveness of endodontic sealers, which may limit the generalizability of the findings [13, 16].

Our findings, as shown in Tables 1-2 and Figure 1, reveal that the selected sealers demonstrate significantly different antibacterial inhibitory effects depending on the root canal sealer type and bacterial strains tested (P<0.01), which is in line with previous research [1,2]. Shantiaee et al. reported that the antibacterial effectiveness of AH26 sealer was notably higher than Apexit and pure ZOE at all selected periods (P<0.001)[12], a result that aligns with our study. Variations in microorganism strains and testing methods may account for these discrepancies. Moreover, agar diffusion has been identified as a significant source of variation in the findings of many studies [17].

Our data showed that the AH26 sealer had the largest bacterial inhibition zone compared to other sealers (P<0.01), as seen in Figure 1. Earlier studies reported similar outcomes [1, 12, 18]. In contrast, some studies [13,19] found that AH26 sealer demonstrated lower or no antimicrobial effects. Siqueira et al. suggested that bisphenoldiglycidyl ether, a known mutagenic component, might be related to the antibacterial efficiency of resin-based sealers [20]. Previous research indicated that formaldehyde production during the polymerization process could enhance the antibacterial capabilities of sealants [21,22]. Eldeniz et al. reported that the AH26 sealer was highly effective against bacteria, with substantial bacterial inhibition after 72 hours, followed by a decrease in the inhibition zone [23]. In contrast to our findings, another study observed no antimicrobial effect for the AH26 sealer on the fifth day of testing [24].

Our results showed an increase in the bacterial inhibition zone for the AH26 sealer at 72 hours and 120 hours, with similar values at 168 hours, as depicted in Figure 1. These findings align with previous research [4, 12]. Zhang et al. noted that AH plus sealer had no significant antibacterial efficacy during the first hour of the experiment. Therefore, the most suitable sealers for root canal treatments should be selected based on their different properties and antibacterial effectiveness. Several experiments confirmed that root canal sealers with highly antibacterial effectiveness were repeatedly found to cause adverse effects during and after treatment and were cytotoxic and mutagenic [13]. Some research has shown that sealers with materials that spread easily produce larger zones of bacterial inhibition [25,26]. Calcium hydroxide [Ca(OH)2] possesses alkaline antimicrobial qualities, which are desirable for therapeutic sealers [2]. The antibacterial effectiveness of calcium hydroxide-containing substances depends on ionization, which releases hydroxyl ions that increase the pH. A previous study reported that a pH>9 leads to irreversible enzyme disruption in the cell membranes of microorganisms, resulting in the loss of biological activity of the cytoplasmic membrane. Additionally, it may cause the destruction of phospholipids or unsaturated fatty acids, leading to a loss of cytoplasmic membrane integrity [21]. However, the pH of sealers alone cannot explain their antibacterial effectiveness [13].

According to ADT, the inefficiency of some calcium hydroxide-based sealers may be due to the lower solubility and diffusion in agar. Sealers containing calcium hydroxide are effective at removing bacteria, with their antibacterial property stemming from the ionization process that releases OH- ions and increases pH.Apexit Plus is a calcium hydroxide-based sealer with bio-equilibrium properties, easy flow for adapting well to complex canals, minimal dimensional change, and low solubility for a good, permanent root canal closure [2]. Apexit displayed the lowest antibacterial effectiveness against *S. aureus* and no antibacterial effectiveness against *K. rhizophila* (Figure 1). These results are consistent with previous studies [1, 23]. M. Zhang et al. concluded that Apexit sealer releases calcium hydroxide exhibits slight toxicity in the fresh state, is ineffective against obligate anaerobic bacteria (*P. melaninogenicus*), and has weak antibacterial effectiveness, especially with *E. faecalis* compared to six other sealers [13]. The low concentration of OH- ions and bacteria's resistance to the alkaline environment provide a simple explanation for these results. The weak antimicrobial effectiveness of Apexit sealer against anaerobic bacteria frequently present in infected root canals has led to its underutilization in treatments. Also, one study [13] noted that fresh iRoot SP killed *E. faecalis* in 2 minutes; AH plus in 5 minutes; EndoRez in 20 minutes; Sealapex and Epiphany in 60 minutes, while freshly mixed Apexit Plus and Tubli Seal failed to kill *E. faecalis* in 60 minutes. However, according to the one-day and three-day samples, the same study showed that iRoot SP and EndoRez had the strongest antibacterial effectiveness, followed by Sealapex and Epiphany, whereas Tubli Seal and AH Plus did not exhibit any significant antibacterial effectiveness. These results are inconsistent with our current findings, where Apexit sealer demonstrated the least antibacterial effectiveness in all samples, as shown in Figure 1. Zinc oxide-based sealers are antimicrobial because they form reactive oxygen species (ROS) and interfere with bacterial membrane proteins[2].

The ZOE sealer showed antimicrobial effectiveness in the inhibition zone of *Staphylococcus aureus, Streptococcus mutans*, and *Enterococcus faecalis* which was higher than many epoxy resin-based sealers [27]. Our data indicated that the zone of inhibition for Pure ZOE sealer in obligate anaerobic culture dishes decreased after 72 hours, while it increased in facultative anaerobic dishes. However, Pure ZOE sealer showed moderate antibacterial effectiveness for both types of bacteria, as shown in Figure 1. High cytotoxicity has been observed with ZOE sealers containing formaldehyde [2, 7, 28]. One study [13] suggested that the antimicrobial effectiveness of root canal sealers containing ZOE is due to the release of free eugenol from the sealer. Eugenol is a phenolic compound active against fungal cells in their vegetative form [29]. Previous studies have shown that ZOE sealer is more suitable for eliminating facultative anaerobic and aerobic bacteria than obligate anaerobic bacteria [2, 4, 12,13]. ZOE sealer has high solubility, which makes it inferior in quality compared to other sealers. It has been frequently demonstrated that ZOE is cytotoxic and that adhesion between GP and ZOE-eugenol is hazardous, especially after combining.

Queiroz et al. indicated that *K. rhizophila* was more effectively inhibited by ZOE sealer, while *E. faecalis* was inhibited by Calen/ZO sealer (p<0.05). Also, S. mutans was inhibited by ZOE, Calen/ZO, and Sealapex with the same intensity (p>0.05) [26], while *E. coli* was most effectively inhibited by ZOE, Calen/ZO, and Sealapex (p<0.05). On the other hand, ZOE sealer and Calen/ZO sealer were equally effective against *S. aureus* (p>0.05), while Sealapex sealer had lower antibacterial effectiveness (p>0.05). EndoRez showed the least antibacterial effectiveness with *S. aureus*, while no antibacterial effectiveness with *K. rhizophila* (P<0.05) (Figure 1). Queiroz et al. noted that EndoREZ sealer has antibacterial effectiveness only against *K. rhizophila* and *S. aureus* [26]. The Calen sealer and Calen/ZO pastes produced inhibition zones greater than 1% CHX with the marker microorganism *E. faecalis* [26], supporting our findings. Also, Queiroz et al. observed that ZO sealer inhibits the growth of *S. sobrinus* and *E. coli*. Inhibition of *E. coli* by both fatty oils and eugenol may explain the fact that the present study produced larger inhibition zones against *E. coli* (23.67 mm diameter) and greater than that of 1% CHX (19.33 mm diameter) [26]. Jones et al. noted that the diameters of the bacterial inhibition zones formed around Apexit sealer against *S. aureus* are larger when compared with the diameters of Calen paste alone or with ZO and Apexit. They concluded that adding ZO sealer to Calen paste had no effect on its antimicrobial effectiveness [30]. *Kocuria rhizophila, Enterococcus faecalis, Streptococcus mutans, Escherichia coli*, and *Staphylococcus aureus*, which are frequently present in endodontic infections, can be arranged in descending order as: ZOE>Calen/ZO>Sealapex>EndoREZ.

Queiroz et al. investigated the in vitro antibacterial effectiveness of primary dental root canal sealers: ZOE, Calen/ZO, Sealapex, and EndoREZ against *Kocuria rhizophila, Enterococcus faecalis, Streptococcus mutans, Escherichia coli*, and *Staphylococcus aureus*, which are frequently present in endodontic infections [26]. They found that the antibacterial efficacy of these sealants followed the descending order: ZOE >Calen/ZO >Sealapex>EndoREZ. While several studies reported a minimal effect of ZOE sealer on the tested bacteria, Apexit sealer exhibited lower antibacterial effectiveness against S. mutans and no effect on *P. melaninogenicus*. Consequently, the sealers were arranged in ascending order of bacterial inhibition zones against S. mutans and *P. melaninogenicus*: AH26 > Pure ZOE >Apexit [1, 7, 26, 30]. These studies reinforce our current findings.

Inadequate closure of the cavity can allow bacterial penetration into the root canal within a few days, potentially leading to persistent apical periodontitis due to residual bacteria and re-infection [31]. Therefore, endodontic fillings should possess antibacterial/antimicrobial properties [29]. The addition of antimicrobial agents to root canal sealers can further enhance their antibacterial properties [19]. In this study, EndoREZ sealer showed the lowest antibacterial effectiveness compared to other endodontic filling materials (Tables 1-2 and Figure 1). Eldeniz et al. [23] noted that EndoREZ, Apexit, and Roeko sealers do not have antibacterial effectiveness according to ADT. However, direct contact test (DCT) results indicated that AH26 and Sultan sealers were strong inhibitors of bacterial growth. They concluded that although EndoREZ sealer exhibited antibacterial properties, it was not as potent an inhibitor of bacterial growth as Sultan and AH26 sealers [23].

Liu et al. reported that EndoREZ sealer containing 0% dimethylaminododecyl methacrylate (DMADDM) showed no antibacterial effectiveness, while sealers containing 1.25% and 2.5% DMADDM demonstrated stability and significant antibacterial effectiveness. They also observed that the antibacterial efficacy of sealers containing DMADDM did not decrease even after 10 days of adjustment, indicating that the addition of DMADDM to EndoREZ could provide long-term antibacterial capabilities (p > 0.1) [1]. This finding suggests that the DMADDM monomer does not dissolve after being combined with EndoREZ, implying that the addition of the DMADDM monomer to EndoREZ might inhibit biofilm growth on the sealer's surface.

After testing the antibacterial effectiveness of seven types of endodontic sealers Apexit Plus, Sealapex, AH Plus, iRoot SP, Tubli Seal, Epiphany SE, and EndoRez, against *Enterococcus faecalis*, it was found that iRoot SP, AH Plus and EndoRez sealers inhibit the growth of *E. faecalis* [13]. This finding aligns with our current study in some aspects but differs in others, as it indicated that only Sealapex and EndoRez demonstrated antimicrobial effectiveness even after seven days of mixing.

G. Geurtsen et al. and Leyhausen suggested that ideal root canal sealers should possess antimicrobial effectiveness and low toxicity to surrounding tissues. They emphasized that dentists should be concerned with biocompatibility, including cytotoxicity, genotoxicity, mutagenicity, and carcinogenicity, among other factors [22, 32]. The antimicrobial effectiveness of root canal sealers is just as important as their chemical and physical properties. Moreover, concerns about drug resistance arise when conventional antibiotics, such as amoxicillin, are added to endodontic sealers [28]. The added antibacterial agent can interact with the sealer and exhibit strong antibacterial capabilities for a long time. Several studies concluded that facultative microorganisms such as *S. aureus, E. faecalis*, and even C. albicans are the most resistant species in the oral cavity and may contribute to root canal treatment failure. Furthermore, the growth and spread of these microorganisms within the canal can destroy periapical tissue, resulting in periapical disease [33,34].

Our findings indicate that distilled water does not possess antibacterial effectiveness for either type of bacteria (Tables 1-2 and Figure 1), consistent with previous research [26]. This study aims to provide dentists with information regarding the quality and properties of these materials. As a result, it is recommended that our findings be considered when selecting root canal sealers, and additional in vivo and in vitro research is warranted. Finally, it is crucial for root canal materials used in primary teeth to possess antimicrobial properties. This is essential to effectively eliminate residual pathogens, neutralize their toxic byproducts, and prevent re-infection of the canal. By doing so, an environment conducive to the healing process can be established and maintained.

## Conclusion

In all selected periods, AH26 sealer demonstrated the largest inhibition zones, while EndoREZ exhibited the smallest inhibition zones for *S. aureus* and *K. rhizophila*. The inhibition zones for both types of bacteria tested were arranged in ascending order as follows: AH26 > Pure ZOE >Apexit/EndoREZ.
